# Stability of and change in substance use risk personality: Gender differences and smoking cigarettes among early adolescents

**DOI:** 10.1016/j.abrep.2021.100360

**Published:** 2021-06-04

**Authors:** J.J.P Mathijssen, A.D. Rozema, M. Hiemstra, M.W.J. Jansen, J.A.M. van Oers

**Affiliations:** a Tilburg School of Social and Behavioral Sciences, Department Tranzo, Tilburg University, the Netherlands; b Academic Collaborative Centre for Public Health Limburg, Public Health Service South Limburg (GGD ZL), Heerlen, the Netherlands; c Department of Health Services Research, CAPHRI Care and Public Health Research Institute, Maastricht University, Maastricht, the Netherlands; d Ministry of Health and Welfare, Den Haag, the Netherlands

**Keywords:** Personality, Early adolescents, Stability, Change, Longitudinal, Smoking behavior

## Abstract

**Objective:**

Adolescents show a steadily increasing inclination toward health risk behaviors, including smoking cigarettes. There is ample evidence that personality traits are related to smoking behavior. However, less is known about the stability of and change in these personality traits during early adolescence and whether smoking behavior affects the developmental trajectories. Moreover, less is known about the influence of gender on the course of personality.

**Method:**

Longitudinal data of three waves were used from 1121 early adolescents. To measure personality, the Substance Use Risk Profile Scale was used. Individual growth curve models were conducted to measure the stability, mean-level change and individual differences in change for personality.

**Results:**

Stability of personality was moderate for boys and ranged from moderate to high for girls. On average early adolescents became more impulsive and more sensation seeking over a period of 18 months. Furthermore, hopelessness for girls increased and the increase in sensation seeking was higher for girls than for boys. Third, smoking behavior was related to all personality traits, indicating that smoking adolescents are more anxious, hopeless, impulsive and sensation seeking than non-smoking adolescents.

**Conclusions:**

Our results are in line with the disruption hypothesis, i.e., during early adolescence there is a dip in personality maturity. There are clear differences between girls and boys in stability of and change in personality traits. Besides, although smoking behavior is related to personality, the change in personality is probably related to other variables.

## Highlights

• During early adolescence, there is a dip in personality maturity.
• There are differences between girls and boys in stability of and change in personality traits.
• Both gender and smoking cigarettes have a small effect on the individual change in personality.

## 1 Introduction

Adolescence is a period of rapid change. Besides brain development and an increasing independence, adolescents show a steadily increasing inclination toward health risk behaviors, including smoking cigarettes (Arnett, 1999, Wellman et al., 2016, Yurgelun-Todd, 2007). Indeed, lifelong health behaviors and habits often begin in adolescence (Alberga et al., 2012, Degenhardt et al., 2008). A significant body of evidence shows that smoking cigarettes among adolescents is related to personality (Ali et al., 2016, Brook et al., 2008, Burt et al., 2000, Krank et al., 2011; Malmberg et al., 2013, Peterson and Smith, 2017, Pokhrel et al., 2014, Quinn and Harden, 2013, Wellman et al., 2016, Woicik et al., 2009), supposing that personality is predictive for smoking behavior. Since especially persistent personality traits seemed to be related to future behavior, it is assumed that personality traits that remain stable over time would be more predictive for smoking behavior.

Research has shown that aspects of personality may change across lifespan (Caspi, Roberts, & Shiner, 2005). There is also evidence that personality changes during adolescence (Borghuis et al., 2017, Van den Akker et al., 2014). In general, there are three different ways in which change can be defined, i.e., (1) differential continuity, (2) mean-level change and (3) individual differences in change (Borghuis et al., 2017; Roberts and DelVecchio, 2000, Roberts et al., 2006). First, differential continuity refers to the extent to which individual differences in a given trait remain steady over time. Differential continuity tends to increase with age. In a comprehensive meta-analysis of longitudinal studies, Roberts and DelVecchio (2000) determined that stability for personality traits was lowest in studies of children, rose to higher levels among young adults, and then reached a plateau for adults between the ages of 50 and 70. Second, mean-level change refers to the extent to which the average values of scores on any given trait within a group rise or fall over time. A meta-analysis by Roberts et al. (2006) has suggested mean-level changes in personality indicating that people become more confident, agreeable, conscientious, and emotionally stable with age. Third, individual differences in change refer to the differences that exist between individuals in the way their personality develops over time (Shek & Ma, 2011).

Personality development across lifespan has been explained in terms of the ‘maturity principle’ (Roberts et al., 2006). This means that people tend to become more agreeable, more responsible and more emotionally stable across life span. However, recently researchers on personality in adolescence are more and more convinced that this principle does not apply to the period of early adolescence. Actually, although only few studies are available, these studies indicate that the disruption hypothesis is more applicable (Ibáñez et al., 2016, Soto et al., 2011). According to this hypothesis, the biological, social, and psychological transitions from childhood to adolescence are accompanied by temporary dips in personality maturity (Soto & Tackett, 2015). For example, results of the study of Soto et al. (2011), showed that, contrary to the ‘maturity principle’, levels of extraversion, agreeableness, conscientiousness, and openness decreased across early adolescence for both boys and girls, whereas neuroticism, increased for girls.

Furthermore, personality change during adolescence has been explained in terms of the dual systems model, which posits that adolescent behavioral development is shaped by the developmental asymmetry between neurobiological systems, i.e., the cognitive control system and the socio-emotional system (Casey & Jones, 2010). The cognitive control system has a prolonged course throughout adolescence, resulting in slow but steady gains in impulse control. By contrast, the socio-emotional system develops rapidly in early adolescence, resulting in a spike in sensation-seeking behavior. This developmental asymmetry between these two systems is thought to drive the rise in risk-taking behavior that is typical of adolescence.

These divergent patterns of personality change in adolescence have been documented by different studies (Harden and Tucker-Drob, 2011, Lynne-Landsman et al., 2011, Shulman et al., 2015, Steinberg et al., 2008). Consistent with the dual systems model, impulsivity seems to decline across adolescent development while sensation seeking initially increases during early adolescence, but then declines during the transition into adulthood. Moreover, Harden and Tucker-Drob (2011) demonstrated that, in addition to mean-level patterns of change, there are individual differences in the pace of change in impulsivity and sensation seeking across adolescence.

Although there is some evidence that gender differences exist in the course of personality development in adolescence (Canals et al., 2005, Kawamoto and Endo, 2015, Shulman et al., 2015), relatively few studies have focused on these differences. This is unfortunate given the differences between boys and girls during adolescence, including neurobiological changes and vulnerability to risk behavior (Dir et al., 2017, Hammerslag and Gulley, 2016). Actually, it has been widely recognized that boys smoke more than girls, both conventional cigarettes and e-cigarettes, (e.g. Grunberg et al., 1991, Pineiro et al., 2016, Statistics Netherlands, 2019; Kong, Kuguru, & Krishnan-Sarin, 2017).

The aim of the present longitudinal study is threefold. First, both the stability of and change in the four smoking related personality traits are studied, i.e., anxiety sensitivity, hopelessness, impulsivity and sensation seeking, among early adolescent boys and girls over a period of 18 months. Second, the influence of gender on the course of personality during early adolescence is studied. Finally, the associations between smoking behavior and changes in personality are studied.

## 2 Methods

### 2.1 Procedure and participants

The data for this study were collected as part of a broader study (Rozema, Hiemstra, Mathijssen, Jansen, & van Oers, 2018). A questionnaire conducted in 2014 mapped out the smoking policies in place at 919 Dutch secondary schools. A total of 77 schools were randomly contacted by telephone and asked whether they would participate. Sixteen schools agreed to participate. Questionnaire data of the adolescents were collected during school hours. In all three waves, adolescents filled out online or paper questionnaires at school under the supervision of a teacher. The baseline measurement took place between March 2014 and May 2015, the 6-month follow-up between September 2014 and November 2015, and the 18-month follow-up between October 2015 and January 2017. In this study, only students who at baseline were in grade 7 to grade 9, whose school participated in all three waves, and who personally participated in each of the three waves were included (N = 1113).

Informed consent was obtained from all participating students. Beforehand, as students were of minority age (<18 years), their parents were fully informed about the study. Some parents (*n* = 30) refused to permit their child’s participation, and these students did not participate in the study. The study was approved by Ethics Review Board of Tilburg University (EC-2014.19).

### 2.2 Measures

*Personality Profiles* were measured using the Dutch translation of the Substance Use Risk Profile Scale (SURPS) (Malmberg et al., 2010, Woicik et al., 2009) at each wave. The SURPS measures four personality traits: anxiety sensitivity, hopelessness, impulsivity, and sensation seeking. Each trait was measured with 5–7 items (in total 23 items) that could be answered on a 4-point scale ranging from 1 = strongly agree to 4 = strongly disagree. Sum scores were calculated per personality trait by summing the answers on the questions. For the three waves, the Cronbach’s alphas were respectively 0.72, 0.77, 0.80 for anxiety sensitivity, 0.86, 0.86, 0.88 for hopelessness, 0.69, 0.74, 0.77 for impulsivity, and 0.69, 0.72, 0.74 for sensation seeking. Reliability and validity of the instrument are adequate (e.g., Krank et al., 2011, Malmberg et al., 2010).

*Smoking behavior.* At each wave*,* adolescents were asked to report, on a 3-point scale, which stage of smoking conventional cigarettes applied to them. Response categories ranged from 1 ‘smoked in the past seven days’, 2 ‘did not smoke in the past seven days’, 3 ‘never smoked. Adolescents who ‘never smoked a cigarette’ and ‘who did not smoke in the past seven days were coded as ‘non- user’ and ‘who smoked in the past seven days were coded as ‘user’. Besides, adolescents were asked the following question ‘In the past four weeks, how many times did you use e-cigarettes?’ The adolescents who did not use e-cigarettes were coded as ‘non-users’, the adolescents who did use one or more times e-cigarettes were coded as ‘users’.

### 2.3 Statistical analyses

Descriptive statistics, attrition analyses, and linear mixed models were conducted using SPSS 24.0. With linear mixed models, individual growth curves (ICG) can be estimated. This means that both within-person changes and between-person developments across time are studied. The unconditional mean model is used to assess the mean of the outcome variable and the amount of outcome variation that exists at intra- and inter-individual levels (Shek & Ma, 2011). This is a one-way ANOVA model with a random effect, with no predictors. It serves as a baseline model to examine individual variation in the outcome variable without regard to time. This model assesses (1) the mean of the outcome variable and (2) the amount of outcome variation that exists in intra- and interindividual levels. Moreover, the proportion of the total outcome variation that is related to interindividual differences can be examined (i.e. the intraclass correlation coefficient (ICC). This is a measure of average autocorrelation of the outcome variable over time, i.e. stability over time.

Our data had a hierarchical structure, with students (first level) nested within schools (second level). That is why we tested in an unconditional linear growth curve model whether it was necessary for our analyses to control for school effect (Shek & Ma, 2011). Using the intra-class correlation coefficient (ICC), clustering effects of schools for the four personality factors were calculated. The ICC is the proportion of the total variation in personality that is attributable to differences between schools. The ICCs ranged from 0.01 (anxiety), 0.03 (impulsivity and sensation seeking) to 0.05 (hopelessness). These results indicate that less than 5% of the variation in the personality variables is due to differences between schools. Since, in general, correcting for a multilevel effect is necessary if the proportion of explained variance is 25% or higher, multilevel modeling for school effects proved not necessary (Heinrich & Lynn, 2001).

The average stability for the four personality traits was calculated by means of the ICC at the individual level. In this case, the ICC is the ratio of the between-individuals variance to the total variance. The ICC can be interpreted as the correlation among observations within the same person, i.e., stability, with a high score indicating a high stability.

The mean-level change and individual differences in change were estimated by means of individual growth curve models for four personality variables. In these models, the mean estimates of the intercept and slopes represent the mean personality score over the three waves and the mean rate of linear change over a period of 18 months, respectively. One of the strengths of IGC is that it allows the irregularity of spacing of waves. In the present study data collection was scheduled at six and eighteen months after the baseline data collection. Therefore, the variable time was added in this model to test its’ linear effect with time = 0 at Wave 1 time = 0.5 at Wave 2 and time = 1.5 at Wave 3.The variance estimates (random effects) of the intercept and the slope represent the variance of the individual growth trajectories around the mean growth trajectory, and indicate the degree of between-person variability around the mean group intercept and the mean group slope.

Linear mixed models were used to assess the association between gender and smoking behavior and (changes in) personality.

## 3 Results

### 3.1 Sample characteristics

**Fig. 1:**
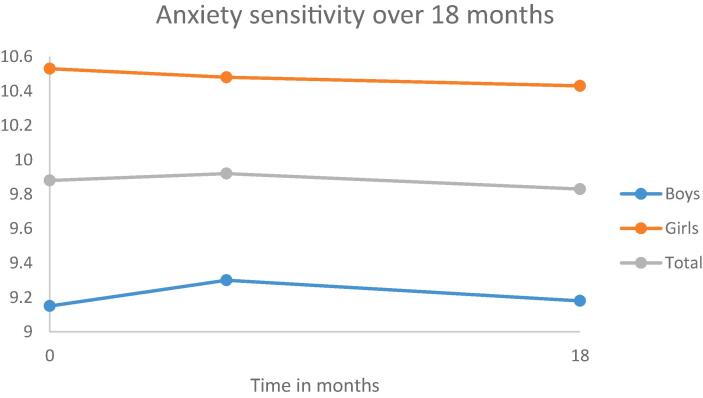
Development of anxiety sensitivity over a period of 18 months.

**Fig. 2:**
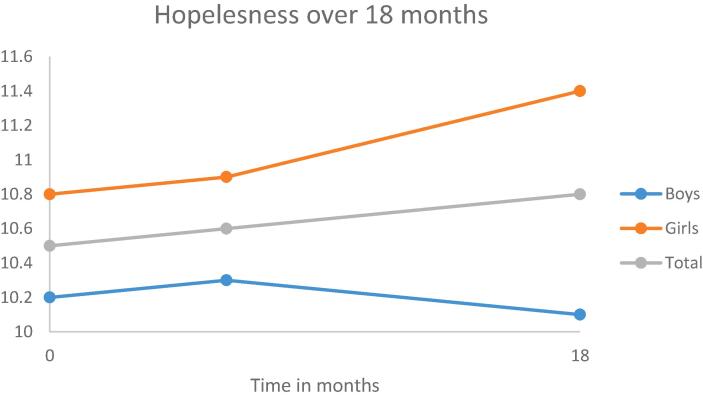
Development of hopelesness over a period of 18 months.

**Fig. 3:**
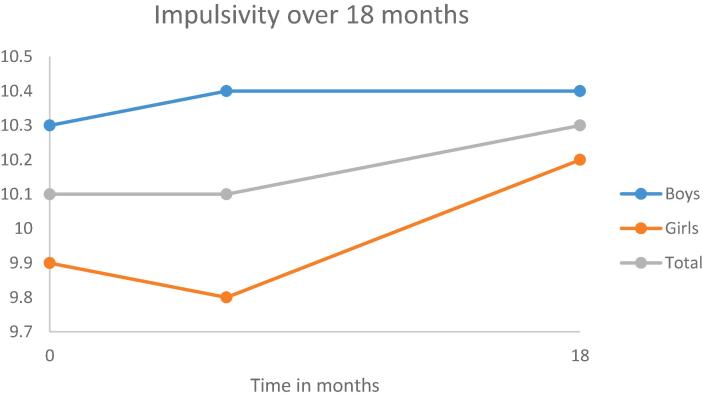
Development of impulsivity over a period of 18 months.

**Fig. 4:**
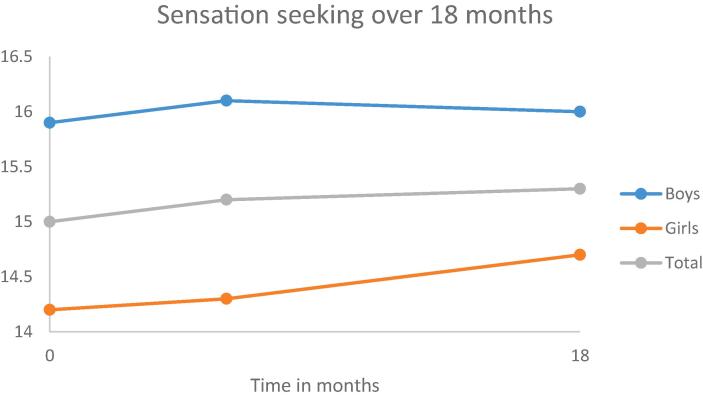
Development of sensation seeking over a period of 18 months.

**Fig. 5:**
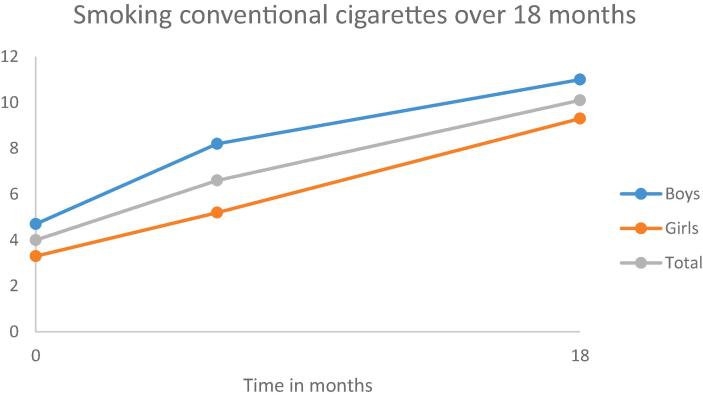
Development of smoking conventional cigarettes over a period of 18 months.

**Fig. 6:**
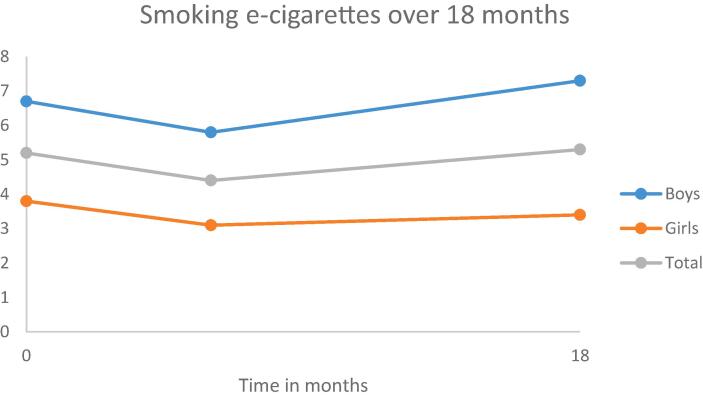
Development of smoking e-cigarettes over a period of 18 months.

Most of the adolescents (n = 956) were of Dutch origin (85.3%). Adolescents’ mean age at baseline was 13.4 (*SD* = 0.9) years and 47.5% were boys. Regarding educational type at baseline, 43.3% of the youths attended lower education (i.e., pre-vocational secondary education), 25.2% attended below average education (i.e., lower general secondary education), 14.7% attended above average education (i.e., higher general secondary education), and 16.8% attended higher education (i.e., pre-university education). At baseline, the participating adolescents were in grade 7–9 (7th grade 47.6%; 8th grade 41.6%; 9th grade 10.8%). Information about the developments of personality traits and smoking behavior over a 18-month period, is given in Fig. 1, Fig. 2, Fig. 3, Fig. 4, Fig. 5, Fig. 6.

Over a period of 18 months recent use of conventional cigarettes increased (OR = 2.15, 95 %CI 1.69–2.73). For the use of e-cigarettes no statistically significant increase or decrease was found. Stability in terms of Kappa was respectively, 0.62 and 0.55 for smoking conventional cigarettes over time for boys and girls. For smoking e-cigarettes the Kappa was 0.37 and 0.34 for boys and girls respectively.

### 3.2 Attrition analyses

Overall, of the 4402 adolescents that participated at T0 (baseline), 1438 (32.7%) did so only at T0; 1442 (32.6%) participated at T0 and T1; 416 (9.4%) participated at T0 and T2; and 1113 (25.3%) participated at T0, T1 and T2. Attrition analysis comparing adolescents that participated at all three measurement moments (*n* = 1113) to the students who participated in only one or two measurements (*n* = 3289) showed that girls were less likely to drop out than boys (χ^2^ (1), = 9.62, *p* ≤ 0.01). Further, the higher grade students were more likely to drop out compared to the lower grade students (χ^2^ (2), = 431.9, *p* ≤ 0.01); and compared to the overall drop-out rate, below average educated adolescents were more likely to drop out and above average and higher educated adolescents were less likely to drop out (χ^2^ (3), = 31.1, *p* ≤ 0.01). No differences were found for anxiety and sensation seeking at baseline between the group who participated in all three waves compared to the group who at least participated in the baseline measurement. Statistically significant differences were found for hopelessness (F (1) = 11.4, p ≤ 0.01) and impulsivity (F(1) = 8.9, p ≤ 0.01), indicating that the drop-outs scored higher on both hopelessness and impulsivity. Finally, the dropouts scored higher on recent tobacco use (9.1%) and the use of e-cigarettes (7.1%), compared to the group who participated in all three waves(respectively 3.9% and 5.1%).After correcting for grade, only the difference in smoking conventional cigarettes, between drop outs and completers was still statistically significant.

### 3.3 Unconditional mean model: stability of personality traits

The ICCs, i.e. the stability over time, computed in the unconditional mean model, (see Table 1, Table 2, Table 3, Table 4) were 0.38, 0.42, 0.44 and 0.56 for respectively anxiety, hopelessness, impulsivity and sensation seeking. These stability coefficients, separately for boys and girls were 0.30 and 0.43 for anxiety sensitivity, 0.33 and 0.48 for hopelessness, 0.40 and 0.49 for impulsivity, and 0.48 and 0.60 for sensation seeking,. This means that between 30% (anxiety) and 60% (sensation seeking) of the total variation is due to inter-individual differences, indicating that the stability of the four personality traits lies between 0.30 and 0.48 for boys and between 0.43 and 0.60 for girls.

**Table 1:** Results of individual growth curves modeling for Anxiety Sensitivity.

	Unconditional mean model	Unconditional growth model	Conditional model with Gender	Conditional model with Gender and Smoking behavior
2 log likelihood (df)	17,098 (3)	17,081 (6)	17,022 (8)	16,975 (10)
Intercept		9.91	10.53	11.98
*Fixed effects*				
**Time** (Mean level change) β (SE)		−0.05 (0.08)	−0.08 (0.12)	−0.09 (0.12)
**Sex**				
Girls			Ref	Ref
Boys β (SE)			−1.32 (0.19)**	−1.36 (0.19)**
Gender*time				
Girls			Ref	Ref
Boys β (SE)			0.07 (0.17)	0.05 (0.17)
**Conventional cigarettes**				
No				Ref
Yes β (SE)				0.55 (0.28)*
**E-cigarettes**				
No				Ref
Yes β (SE)				0.95 (0.29)**
*Random effects* (variances)				
**Residual**	8.04 (0.25)**	7.27 (0.31)**	7.27 (0.31)**	7.28 (0.31)**
**Intercept** (individual level)	5.00 (0.34)**	5.34 (0.50)**	4.91 (0.49)**	4.83 (0.49)**
**Slope time** (individual level)		1.33 (0.43)**	1.33 (0.43)**	1.28 (0.43)**
**Covariance** intercept and slope time		−0.50 (0.36)	−0.48 (0.35)	−0.46 (0.35)

* p ≤ 0.05.

** p ≤ 0.01.

**Table 2:** Results of individual growth curves modeling for hopelessness.

	Unconditional mean model	Unconditional growth model	Conditional model with Gender	Conditional model with Gender and Smoking behavior
2 log likelihood (df)	17,368 (3)	17,339 (6)	17,261 (13)^1^	17,178 (15)^1^
*Intercept*		10.53	10.73	12.96
*Fixed effects*				
**Time** (Mean level change) β (SE)		0.17 (0.09)	0.40 (0.12)**	0.40 (0.12)**
**Sex**				
Girls			Ref	Ref
Boys β (SE)			−0.49 (0.21)*	−0.55 (0.21)**
Sex*time				
Girls			Ref	Ref
Boys β (SE)			−0.49 (0.18)**	−0.51 (0.17)**
**Conventional cigarettes**				
No				Ref
Yes β (SE)				0.54 (0.29)
**E-cigarette**				
No				Ref
Yes β (SE)				1.44 (0.30)**
*Random effects (variances)*				
**Residual**	8.48 (0.26)**	7.25 (0.32)**	7.25 (0.32)**	7.19 (0.32)**
**Intercept** (individual level)	6.04 (0.39)**	7.51 (0.59)**	6.96 (0.57)**	6.86 (0.56)**
**Slope time** (individual level)		2.09 (0.46)**	2.04 (0.45)**	1.88 (0.49)**
**Covariance** intercept and slope time		−1.51 (0.40)**	−1.49 (0.40)**	−1.40 (0.40)**

* p < .05.

** p < .01.

1 Corrected for grade and education level at T0.

**Table 3:** Results of individual growth curves modeling for Impulsivity.

	Unconditional mean model	Unconditional growth model	Conditional model with Gender	Conditional model with Gender and Smoking behavior
2 log likelihood (df)	16,509 (3)	16,489 (6)	16,431 (11)^1^	16,343 (13)^1^
*Intercept*		10.04	8.84	10.96
*Fixed effects*				
**Time** (Mean level change) β (SE)		0.15 (0.07)**	0.23 (0.10)*	0.19 (0.10)
**Gender**				
Girls			Ref	Ref
Boys β (SE)			0.48 (0.18)**	0.43 (0.18)*
Gender*time				
Girls			Ref	Ref
Boys β (SE)			−0.15 (0.15)	−0.17 (0.15)
**Conventional cigarettes**				
No				Ref
Yes β (SE)				1.22 (0.26)**
**E-cigarettes**				
No				Ref
Yes β (SE)				89 (0.27)**
*Random effects (variances)*				
**Residual**	6.34 (0.19)**	5.82 (0.25)**	5.82 (0.25)**	5.87 (0.26)**
**Intercept** (individual level)	5.04 (0.31)**	5.00 (0.43)**	4.60 (0.42)**	4.28 (0.41)**
**Slope time** (individual level)		0.88 (0.34)**	0.87 (0.34)**	0.74 (0.34)*
**Covariance** intercept and slope time		−0.14 (0.29)	−0.10 (0.29)	−0.06 (0.28)

* p ≤ 0.05.

** p ≤ 0.01.

1 Corrected for education level at T0.

**Table 4:** Results of individual growth curves modeling for sensation.

	Unconditional mean model	Unconditional growth model	Conditional model with Gender	Conditional model with Gender and Smoking behavior
2 log likelihood (df)	17,694 (3)	17,680 (6)	17,621 (8)	17,536 (10)
*Intercept*		15.04	14.17	15.67
*Fixed effects*				
**Time** (Mean level change) β (SE)		0.20 (0.08)*	0.36 (0.12)**	0.34 (0.12)**
**Sex**				
Girls			Ref	Ref
Boys β (SE)			1.82 (0.23)**	1.78 (0.23)**
Sex*time				
Girls			Ref	Ref
Boys β (SE)			−0.33 (0.17)*	−0.36 (0.17)*
**Conventional cigarettes**				
No				Ref
Yes β (SE)				0.89 (0.31)**
**E-cigarettes**				
No				Ref
Yes β (SE)				0.66 (0.31)*
*Random effects (variances)*				
**Residual**	8.12 (0.25)**	7.55 (0.33)**	7.54 (0.33)**	7.52 (0.33)**
**Intercept** (individual level)	10.25 (0.56)**	9.96 (0.70)**	9.14 (0.66)**	8.88 (0.65)**
**Slope time** (individual level)		0.96 (0.43)*	0.94 (0.43)*	0.86 (0.43)*
**Covariance** intercept and slope time		0.04 (0.40)	0.18 (0.39)	0.22 (0.39)

* p ≤ 0.05.

** p ≤ 0.01.

### 3.4 Unconditional linear growth curve model

As can be seen in Table 1, Table 2, Table 3, Table 4 (second model), in general, early adolescents become more impulsive and more sensation seeking over a period of 18 months.

The residual variances decreased with 7.0% for sensation seeking till 14.5% for hopelessness. The individual intercept (random effect, see Table 1, Table 2, Table 3, Table 4) was significant for each of the four personality traits, indicating that the score on personality for individual adolescents is statistically different from the average score on personality. Moreover, also all individual slopes for time were statistically significant. This means that the development over time for anxiety sensitivity, hopelessness, impulsivity and sensation seeking differs between individuals. Finally, only for hopelessness a negative statistically significant association was found between the individual intercept and slope. This indicates that adolescents who score low on hopelessness had a higher linear increase over time, whereas adolescents with a high score on hopelessness had a slower linear increase over time.

### 3.5 Conditional linear growth curve model: gender

Controlling for grade at T0 only yielded a statistically significant improvement for the model of hopelessness. Therefore, we corrected for grade n the model for hopelessness,. Besides, adding school type improved the models for hopelessness and impulsivity, therefore school type was added to these two models.

Adding gender to the models yielded statistically significant improvements for all the models. The results are shown in the third model in Table 1, Table 2, Table 3, Table 4. Gender was statistically significant associated with each of the personality traits; indicating that boys scored lower on anxiety sensitivity and hopelessness and higher on impulsivity and sensation seeking than girls. Moreover, girls have a higher increase in hopelessness and sensation seeking over a period of 18 months than boys.

Besides, the random effects remained statistically significant. Actually, between 7.3% and 8.2% of the variance in individual initial status was explained by gender. Moreover, the variances in the individual slopes were explained by 0% (anxiety sensitivity) till 2.4% (hopelessness).

### 3.6 Conditional linear growth curve model: gender and smoking conventional cigarettes and ecigarettes

Both the smoking of conventional and electronic cigarettes were, independently of each other, related to all personality traits (see the fourth model in Table 1, Table 2, Table 3, Table 4). Smoking adolescents scored higher on anxiety sensitivity, hopelessness, impulsivity and sensation seeking. Only the association between smoking conventional cigarettes and hopelessness was not statistically significant. Since smoking behavior is a time-varying covariate, the βs of smoking conventional cigarettes and e-cigarettes demonstrated that a change in smoking behavior was related to change in personality. For example, the β of 1.22 for smoking conventional cigarettes in the model of impulsivity, indicates that the impulsivity score of an adolescent who starts smoking will increase with 1.22 points. After controlling for smoking conventional and e-cigarettes, the increase in impulsivity was not statistically significant anymore. The residual variances remained more or less the same after including smoking cigarettes to the models. Moreover, the decrease of the variance of the individual slopes for personality, indicates that respectively 3.8%, 7.8%, 14.9% and 8.5% of the difference between individuals in the course of anxiety sensitivity, hopelessness, impulsivity and sensation seeking can be attributed to smoking cigarettes.

## 4 Discussion

The present study tested the stability of and the change in smoking related personality traits during early adolescence and whether gender and smoking behavior affects the course of personality during an 18-month period in early adolescence.

First, in general the stability of the personality traits is moderate for boys (ranging from 0.30 to 0.48) and moderate to strong for girls (ranging from 0.43 anxiety sensitivity to 0.60 for sensation seeking). Second, statistically significant changes in mean levels were found for two personality traits, indicating that early adolescents become more impulsive and sensation seeking over a period of 18 months. Moreover, compared to boys, girls showed an increase in hopelessness and a higher increase in sensation seeking. Third, there is also a difference in the development over time between the adolescents for all personality traits. This means, for example, that some adolescents will become more anxious over time, whereas other adolescents become less anxious. Actually, these individual differences remained even after correcting for gender and smoking cigarettes. Fourth, smoking behavior is related to all personality traits. Both smoking conventional and e-cigarettes were, independently from each other, related to higher scores on anxiety, hopelessness, impulsivity and sensation seeking.

### 4.1 Stability

A meta-analysis performed by Roberts and DelVecchio (2000) reported an average stability coefficient of 0.43 for personality among adolescents. Our results are more or less in line with this study, with lower stability scores for boys. The studies that examined the stability of the substance use-related personality traits reported the same stability as we found for girls (about 0.50) (e.g. Malmberg et al., 2013). However, the stability we found for boys (about 0.33) was significantly lower. Since stability of personality increases with age, one possible explanation for the higher stability in personality of girls is that they mature earlier than boys (Klimstra, Hale, Raaijmakers, Branje, & Meeus, 2009). The higher stability for the more externalizing traits (impulsivity and sensation seeking) compared to the more internalizing traits (anxiety sensitivity and hopelessness) is in line with previous studies (Krank et al., 2011, Malmberg et al., 2013). Moreover, this difference in stability possibly indicates that symptoms of anxiety sensitivity and hopelessness seemed to be more episodic in time.

In general, the moderate stability estimates indicate that changes in personality exist at the rank-order level. This means, for example, that an adolescent who scores high on anxiety sensitivity at baseline, compared to other adolescents, will not automatically score relatively high on anxiety sensitivity 18 months later. Since the predictive power of personality for future behavior is dependent on its’ stability (Wu, 2020) the relatively low stability for anxiety sensitivity indicates that this personality trait is probably a less suitable predictor for future behavior. On the other hand, the relatively high stability for sensation seeking, especially for girls, makes it likely to be a good predictor. Moreover, given the increase in stability in personality from childhood to young adulthood (Roberts & DelVecchio, 2000), the found moderate stability over a 18 month period in our study indicates that early adolescence is a specifically formative stage for personality that need more attention in future studies.

### 4.2 Mean level change

Our results seem to correspond partly with the disruption hypothesis that during early adolescence there is a dip in personality maturity (Soto and Tackett, 2015, Van den Akker et al., 2014). Contrary to the maturity principle of a decrease in anxiety sensitivity, hopelessness, impulsivity and sensation seeking, we find no change in anxiety sensitivity and hopelessness but an increase in both impulsivity and sensation seeking. At the same time, our results seem contradictory to the dual systems model, which states that patterns of development in impulsivity and sensation seeking during adolescence are distinct, i.e., there is a steady decrease in impulsivity and an increase in sensation seeking during early adolescence. However, it is important to realize that the increase in impulsivity over time was not statistically significant anymore after controlling for smoking conventional and e-cigarettes. Since smoking conventional cigarettes both increases over time and is related to impulsivity, the initial increase we found for impulsivity is probably related to the change in smoking conventional cigarettes. Actually, the onset of smoking conventional cigarettes is accompanied by an increase in impulsivity with 1.22 points. Moreover, a study of Treur et al. (2015) demonstrated that smoking during adolescence was associated with an increase in attention problems, a part of impulsivity, in adulthood, suggesting that smoking during adolescence influences the brain which in turn leads to more attention problems. Further research is urgently needed to understand this result.

The increase in sensation seeking was higher for girls than boys. Though girls are, on average, still less sensation seeking than boys. This corresponds with the meta-analysis of Cross, Cyrenne, and Brown (2013) in which it was demonstrated that across lifespan men score higher on sensation seeking than women. Since sensation seeking among adolescents is a complex interplay of developmental influences that run via gender-specific processes (Harden et al., 2018), more research is needed to get insight in the developmental processes of sensation seeking during adolescence.

The finding that girls showed a higher increase in hopelessness across time, together with the finding that girls already score higher than boys on this trait, implies that the difference between the two genders is already manifest in early adolescence, and increases over time. This is in line with previous studies that demonstrated that girls are more likely to develop internalizing problems during adolescence, concurrent with pubertal development (Paus, Keshavan, & Giedd, 2008).

### 4.3 Individual differences in change

Comparing the residual variances between the unconditional mean model and the unconditional growth model demonstrated that 7% (sensation seeking) to 15% (hopelessness) of the within individual variation was associated with linear rate of change. Although adding time provided a better fit of the model, the effects were rather small. The significant random effects (i.e. the intercept and slope) indicate that there are individual differences both in the initial status of the personality traits and in linear change of personality over time. This means that the mean level changes we found provides a less accurate description for individuals’ change in personality traits. This is in accordance with what Borghuis et al. (2017) found for extraversion and emotional stability. Moreover, the correlation between the intercept and linear growth parameter on hopelessness was statistically significant, indicating that adolescents who score higher on hopelessness experienced a slower increase over time, whereas adolescents with lower scores experienced a faster linear growth over time.

After including gender in the models, the random effects remained statistically significant. Actually, the variances in the individual slopes were explained by maximum of 2.4%. Also after including smoking conventional and e-cigarettes the random effects remained statistically significant. Only between 4% and 15% of the variance in individual changes of personality could be explained by smoking cigarettes, indicating that probably other factors are responsible for the change in personality. Since early adolescence is an important developmental period in which young people show a steadily increasing inclination toward health risk behaviors, including alcohol and drug use, these are also important factors to study. Moreover, also situational and environmental factors, such as life events, which have demonstrated to be related to changes in personality (Bleidorn, Hopwood, & Lucas, 2018) need to be included in future studies.

### 4.4 The association of smoking behavior with (the course of) personality

Smoking behavior, both conventional and e-cigarette use, is related to personality. After controlling for gender, smoking adolescents scored higher on anxiety, hopelessness, impulsivity and sensation seeking.

The few studies on the effects of smoking on personality change were predominantly conducted in adult samples. The associations found in these samples may be explained by health-related pathways (Stephan, Sutin, Luchetti, Caille, & Terracciano, 2019). For example, smoking is related to a greater likelihood of depressive symptoms (Payne, Ma, Crews, & Li, 2013) and to a deterioration in physical health (Lim et al., 2012). Poor health, in turn, is associated with higher neuroticism, and lower extraversion, openness, agreeableness, and conscientiousness over time (Jokela, Hakulinen, Singh-Manoux, & Kivimäki, 2014). Since adolescents do not yet experience health related problems as a consequence of smoking, the association between smoking and personality change may not yet be visible. There are also indications that changes in smoking status are related to changes in personality, indicating that quitting smoking is related to decreases in impulsivity and neuroticism (Littlefield & Sher, 2012). Although smoking conventional cigarettes steadily increases during early adolescence, there are probably no smoking quitters yet.

This is one of the first longitudinal studies to evaluate differences between boys and girls regarding the stability in and change of smoking behavior-related personality traits in a large representative sample of adolescents. However, the study has certainly some limitations. First, we had substantial missings; however, the missings were probably random. Since it was the teacher who decided whether or not his class should fill out a questionnaire, the dropout rate did not seem to depend on characteristics of the adolescents themselves. Yet, we found some differences between the dropouts and participants, indicating that the results should be regarded cautiously. However, we can conclude that there are striking differences between the genders, making it valuable to consider gender differences in smoking-related personality traits (Ali et al., 2016). Second, as we conducted only three waves, it was not possible to establish an exact shape for mean-level change. Third, the measurement of smoking conventional cigarettes was fairly crude. We asked whether the adolescents have smoked in the past seven days. Although we see a clear increase in smoking cigarettes over a period of 18 months, especially for early adolescents, who are generally not yet daily smokers, it would probably be more informative to ask for smoking in the past four weeks. Fourth, we studied whether it was necessary to control for grade and education level, however there are possibly other confounders that have an association with the course of personality, such as life events and other risk behaviors.

In conclusion, the present findings suggest that, during early adolescence, different personality traits related to smoking have, in general, a moderate stability. Especially anxiety sensitivity and hopelessness are probably less suitable for predicting future behavior. Our results are in line with the disruption hypothesis that during early adolescence there is a dip in personality maturity, both hopelessness for girls and impulsivity and sensation seeking for girls and boys increase during early adolescence. However, the increase in impulsivity was at least partly due to the increase in smoking behavior. Moreover, the smoking-related personality traits, hopelessness and sensation seeking, seem to have a different trajectory for boys and girls. Although both gender and smoking are related to the level of personality, they were only minimally related to the differences between individual early adolescents in initial status and change. So more longitudinal studies are clearly necessary to get more insight in the trajectories of personality and its associations.

## Funding

This study was funded by the Netherlands Organization for Health Research and Development (ZonMw) and the National Institute for Public Health and the Environment (RIVM). The funder had no role in the design of the study, in the collection, analysis, or interpretation of data, or in the writing of the manuscript. The funder has no access to data collected.

## Declaration of Competing Interest

The authors declare that they have no known competing financial interests or personal relationships that could have appeared to influence the work reported in this paper.
